# Validity of food and nutrient intakes assessed by a food frequency questionnaire among Chinese adults

**DOI:** 10.1186/s12937-024-00921-9

**Published:** 2024-02-27

**Authors:** Dong Zhao, Yiying Gong, Liyan Huang, Rongxia Lv, Yuxuan Gu, Chunxiao Ni, Dafang Zhu, Min Yang, Shuang Rong, Ronghua Zhang, Changzheng Yuan

**Affiliations:** 1 Department of Nutrition and Food Safety, Zhejiang Provincial Center for Disease Control and Prevention, Hangzhou, Zhejiang 310051 China; 2 School of Public Health, the Second Affiliated Hospital, Zhejiang University School of Medicine, Hangzhou, Zhejiang 310058 China; 3 Department of Social Security, Nanjing Normal University, Nanjing, Jiangsu 210023 China; 4 National Institute for Nutrition and Health, Chinese Center for Disease Control and Prevention, Beijing, 100050 China; 5 Department of Nutrition, School of Public Health, Wuhan University, Wuhan, 430071 China; 6 Research Center of Public Health, Renmin Hospital of Wuhan University, Wuhan, 430060 China; 7 Department of Nutrition, Harvard T.H. Chan School of Public Health, Boston, Massachusetts 02115 USA

**Keywords:** Food frequency questionnaire, 24-hour dietary recall, Validity, Food composition table, Chinese adults

## Abstract

**Background:**

Studies regarding the validity of the food frequency questionnaire (FFQ) and the food composition table (FCT) are limited in Asian countries. We aimed to evaluate the validity of a 64-item FFQ and different methods of constructing the FFQ FCTs for assessing dietary intakes of foods and nutrients among adults in eastern China.

**Methods:**

A total of 2325 participants (aged 56.2 ± 14.9 years, 51.6% female) from nine cities in Zhejiang province who completed a 64-item FFQ and 3-day 24-hour dietary recalls (24HRs) in 2015 were included. Eight FFQ FCTs were generated covering food items and specific weights estimated using professional knowledge, representative 24HRs data, or the Chinese FCT (CFCT). Energy-adjusted intakes of foods and nutrients were estimated by residual and energy density methods. Spearman correlation coefficients (SCCs) of intakes of 14 food groups and 17 nutrients between FFQ and 24HRs were calculated to evaluate the overall validity of FFQ.

**Results:**

The average intakes of most food groups and nutrients assessed with FFQ were higher than those assessed using the 24HRs. For the food groups, the averaged energy-adjusted (residual method) SCC between FFQ and 24HRs was 0.27, ranging from 0.14 (starch-rich beans) to 0.49 (aquatic products). For nutrient assessment, the weighted FCT (WFCT) performs the best, and the averaged energy-adjusted (residual method) SCC was 0.26, ranging from 0.16 (iron) to 0.37 (potassium). Similar correlations with 24HRs were observed when using other FFQ FCT in the calculation of nutrient intakes.

**Conclusion:**

The 64-item Chinese FFQ and the WFCT were reasonably valid to assess the dietary intakes of certain foods and nutrients among adults in eastern China.

**Supplementary Information:**

The online version contains supplementary material available at 10.1186/s12937-024-00921-9.

## Introduction

The food frequency questionnaire (FFQ), with the merits of practical efficiency and economic feasibility, is one of the primary methods to assess long-term dietary consumption in large-scale epidemiological studies [1–3]. A recent meta-analysis among healthy adults including 130 validation studies worldwide demonstrated that the mean correlation coefficients of nutrients assessed by FFQs was 0.416 compared with the 24-hour dietary recalls (24HRs), and was 0.373 compared with dietary records (DRs), indicating a moderate-to-high validity of using FFQs to assess nutrient intakes, despite some low performance for several specific nutrients [4]. In another meta-analysis among Chinese free-living adults across 30 validation studies, the pooled correlation coefficients were 0.44 for nutrients and 0.41 for food groups compared with 24HRs; and the corresponding correlation coefficients were 0.35 for nutrients and 0.42 for food groups compared with DRs [5].

Although the validity of FFQ for measuring long-term dietary intakes has been evaluated extensively in many studies, the application of FFQ in population-based cohort studies of nutritional factors and chronic diseases is still restricted in countries (e.g., China) with diversified dietary cultures. In addition, food composition table (FCT) designed for a specific FFQ is usually used to calculate a variety of nutrient intakes based on the structured list of FFQ food intake information [6]. For example, the 24HRs data has been used to calculate the food consumption ratio and ascertain the weights in the study population, the corresponding FFQ FCT was developed and then utilized to calculate the overall nutrients intakes [6, 7]. However, very few studies have described the standard procedure of developing corresponding FFQ FCT for nutrient calculation, which may also hinder the extended use of FFQ across different populations.

To address these issues, the present study aimed to evaluate the validity of a quantitative FFQ and the different methods of constructing FFQ FCTs for assessing dietary intakes of foods and nutrients among adult residents in eastern China.

## Methods

### Study design and population

**Fig. 1:**
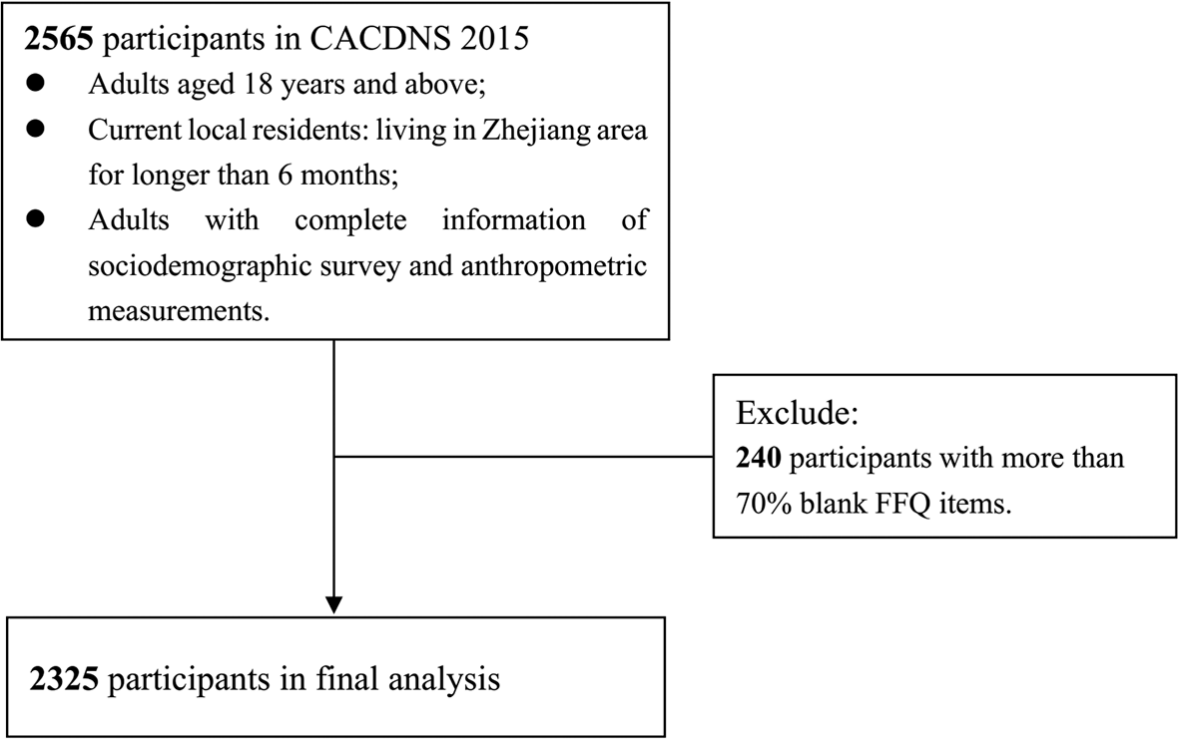
Participant flow chart

The data were derived from the 2015 China Adult Chronic Disease and Nutrition Surveillance (CACDNS 2015), which was organized by the National Institute of Nutrition and Health, Chinese Center for Disease Control and Prevention. A multistage stratified cluster random sampling method was carried out to sample 302 monitoring sites across 31 provinces in China mainland [8–10]. The detailed description has been published elsewhere [11]. The present study collected data from 10 monitoring sites of the Zhejiang Province, including Hangzhou, Ningbo, Wenzhou, Jiaxing (including 2 monitoring sites), Shaoxing, Jinhua, Quzhou, Taizhou, and Lishui. Investigations were performed from August to November 2015 among 2565 adults currently residing in the Zhejiang area for longer than 6 months. The project collected dietary survey, sociodemographic inquiring survey and anthropometric measurements of each participants. Weight and height were used to calculate body mass index (BMI, calculated as weight divided by the square of height (kg/m^2^)) [9]. The dietary survey including a 64-item quantitative food frequency questionnaire (FFQ), and 3-day 24HRs with in-person interviews [12]. After excluding 240 participants with more than 70% blank FFQ items, 2325 individuals (1126 males and 1199 females) were finally included in the study (Fig. 1). This project was approved by the Ethics Review Committee of the Chinese Center for Disease Control and Prevention (No. 201,519-B). All of the participants enrolled in this study signed the informed consent prior to the start of the study.

### Food frequency questionnaire

FFQ was sourced from the CACDNS 2015 and was constructed based on dietary habits and experiences in China. It consisted of 64 food items and two open questions, grouped into 12 categories: staple foods (9 food items), legumes (5 food items), vegetables (3 food items and one opening question), mushrooms (5 food items), fruits (2 food items and one opening question), dairy products (6 food items), meats (7 food items), aquatic products (5 food items), eggs (3 food items), desserts and snacks (8 food items), beverages (5 food items), and alcohol (6 food items). Participants were asked to report the average quantity and frequency of food intake that they consumed in the previous 12 months. For each food item, participants were asked to answer how frequently (the number of times per day, per week, per month, per year, or never) they consumed the food. The quantity of each food item they consumed each time was measured in grams or milliliters. The two open questions were regarding the top five fresh fruits and vegetables that participants consumed the most frequently in the previous year during the winter-spring and summer-autumn seasons, respectively.

### Consecutive 3-day 24-hour dietary recalls

Consecutive 3-day 24HRs, including two weekdays and one weekend day, was performed during the same period as FFQ. Each participant had an in-person interview with a trained interviewer after dinner. During the interview, participants were asked to recall all the foods and drinks that they consumed in the 24h preceding the survey. A booklet containing measuring guides for commonly operational kitchen utensils, such as bowls and plates, were used for assistance of food quantity recall. The seasonings were weighted using a food scale once by the end of the day at the household level and the individual condiments consumption was calculated based on the energy proportion and the number of meals consumed at home [13, 14].

### Foods and nutrient intake assessment

Fifteen food groups and seventeen nutrients were analyzed and presented in this study. The foods consumption was calculated using the following formula: frequency multiplied by the quantity of the foods in the corresponding food items. As for the individual daily nutrient intake, we established eight converted FFQ FCTs based on professional knowledge, representative 24HRs data, or the 2002 CFCT [15]. Six of the eight converted FCTs that we developed were based on the 24HRs data and according to a different constituent for each food item listed in the same food group. For example, as for the weighted FCT method (WFCT) (we generated FCT with a weighted mean of the top 80% of food items by intake amount), we first ranked the food items in accordance with their intake amount from high to low based on 24HRs data obtained for each food group; we then calculated the converted FCT for each food group by selecting food items contributing to 80% of the total amount and weighted by their constituent ratios. Further, the similar calculation method was used to derive the other five type of FFQ FCTs (the arithmetic mean of the top 80% of food items by intake amount/intake frequency; the weighted mean of the top 80% of food items by intake frequency; the arithmetic mean of the top 10 food items by intake amount/intake frequency) (Supplementary Table 1). Furthermore, we also established two converted FCTs that were not based on the 24HRs data. One was the reference method, calculated for each food group based on the food items provided in previous studies with similar dietary cultures to the Zhejiang residents [6, 7, 16, 17]. And the other one was the ‘all food items’ method, calculated for each food group based on the arithmetic mean of all food items in the 2002 CFCT (Supplementary Table 1). Finally, eight converted FCTs were established in this study to calculate nutrient intakes.

### Statistical analysis

Baseline characteristics of participants were presented as mean (standard deviation, SD) for continuous variables and number (percentage) for categorical variables. The daily intakes of nutrients were presented as mean and SDs. The daily intakes of different food groups were additionally presented by median and quartile ranges. The validity of the FFQ was assessed by calculating correlations of the intakes of nutrients and foods using Spearman correlation coefficients (SCCs) with those assessed by 24HRs. To keep the total energy intake constant and maintain the participants’ intakes of specific nutrients when altering the composition of their diet, we used two energy-adjustment methods: residual and energy density. The residual method uses the residuals from the regression of the nutrient intakes on total energy intake with a reference energy level. The energy density method uses the total energy intake to divide the nutrient portion [18].

In the present study, statistical analysis software (SAS) version 9.2 was used. A *p-*value less than 0.05 (two-tailed) was considered statistically significant.

## Results

Among 2325 participants (1126 males and 1199 females), the mean age was 56.2 (SD, 14.9) years and the mean BMI was 23.4 (SD, 3.2) kg/m^2^. Participants were predominantly of the Han nationality (98.8%) and married (90.4%), and 62.4% were educated up to primary school. The subgroups defined by sex (male and female) had similar characteristics to the overall participants. (Table 1).

**Table 1:** Basic characteristics of the study participants, overall and by subgroups of gender

Variable		Overall (*n* = 2325)	Subgroups	
			Male (*n* = 1126)	Female (*n* = 1199)
Age, years ^a^		56.2 ± 14.9	57.2 ± 15.1	55.3 ± 14.8
Height, cm (*n* = 2226) ^a^		159.9 ± 8.4	165.4 ± 6.7	154.8 ± 6.3
Weight, kg (*n* = 2226) ^a^		60.1 ± 10.4	64.1 ± 10.4	56.3 ± 8.9
BMI(*n* = 2226) ^ab^		23.4 ± 3.2	23.4 ± 3.2	23.5 ± 3.3
Nationality ^c^				
Han		2,297 (98.8)	1,116 (99.1)	1,181 (98.5)
Others		28 (1.2)	10 (0.9)	18 (1.5)
Education ^c^				
Primary school and below		1,451 (62.4)	611 (54.3)	840 (70.1)
Middle school		503 (21.6)	296 (26.3)	207 (17.3)
High school		190 (8.2)	125 (11.1)	65 (5.4)
College and above		181 (7.8)	94 (8.4)	87 (7.3)
Marital status ^c^				
Unmarried	84 (3.6)		51 (4.5)	33 (2.8)
Married	2,102 (90.4)		1,034 (91.8)	1,068 (89.1)
Others	139 (6.0)		41 (3.6)	98 (8.2)

^a^ Values are presented as Mean ± SD

^b^ BMI was calculated as weight (kg)/height (m)^2^

^c^ Values in brackets are presented as %

The distributions of the absolute intakes of the food groups are shown in Supplementary Table 2. The mean intakes for most food groups assessed by FFQ were higher than those by 24HRs, except for five food groups including potatoes, starch-rich beans, soybean and its products, red meat and poultry, and aquatic products. When the 24HRs were used as the comparison method, the unadjusted SCC for foods assessed by FFQ compared with 24HRs were lowest for starch-rich beans (*r* = 0.11) and highest for aquatic products (*r* = 0.56), with the mean SCC being 0.30. In addition, the corresponding SCCs were similar after adjustment for energy. The energy-adjusted SCC by the residual method ranged from 0.14 (starch-rich beans) to 0.49 (aquatic products) (mean energy-adjusted *r* = 0.27). Similarly, the energy-adjusted SCC by the energy density method ranged from 0.11 (starch-rich beans) to 0.54 (aquatic products) (mean adjusted *r* = 0.29) (Supplementary Table 3).

Among eight different approaches to construct the FFQ FCT, daily intakes of nutrients assessed by the FFQ and the WFCT method demonstrated the highest correlations with those assessed by 24HRs in the current study. (Table 2, Supplementary Tables 4, 5). In addition, the correlation coefficients of nutrients were generally lower for the FCT method constructed by ‘all food items’ approach.

**Table 2:** Mean values and standard deviations for absolute daily nutrient intakes estimated by FFQ and 24HRs

Nutrient	FFQ				24HRs
	FCT1^a^	FCT2^b^	FCT3^c^		
Total energy, kcal	1,932.0 (1,092.2)	1,891.1 (1,075.5)	1,885.5 (1,062.3)		1,417.6 (493.1)
Protein, g	69.6 (43.7)	66.9 (42.1)	68.6 (43.3)		58.1 (24.4)
Total fat, g	33.8 (30.0)	30.2 (24.8)	34.4 (30.2)		34.5 (23.4)
Carbohydrate, g	324.5 (189.8)	322.2 (189.1)	318.3 (177.3)		218.1 (78.7)
Fiber, g	12.3 (12.9)	10.7 (11.1)	18.5 (14.9)		9.3 (7.5)
Vitamin A, µg	523.3 (359.4)	275.2 (207.1)	205.4 (163.7)		442.7 (520.8)
Vitamin B1, mg	1.2 (1.8)	1.2 (1.9)	1.2 (1.9)		0.7 (0.4)
Vitamin B2, mg	1.8 (8.0)	1.9 (8.9)	2.0 (8.9)		0.7 (0.3)
Vitamin C, mg	93.8 (71.4)	92.4 (86.7)	52.1 (70.9)		70.6 (47.1)
Calcium, mg	522.6 (399.5)	425.1 (314.2)	392.1 (353.8)		405.4 (206.5)
Phosphorus, mg	1,025.3 (613.7)	900.1 (530.2)	930.7 (579.1)		820.4 (309.6)
Potassium, mg	1,816.5 (1,215.6)	1,656.6 (1,159.3)	2,227.1 (1,419.4)		1,463.1 (695.6)
Sodium, mg	1,014.3 (1,179.5)	775.1 (1,133.5)	1,236.6 (1,485.4)		745.6 (686.4)
Iron, mg	22.3 (22.6)	20.0 (20.1)	28.8 (37.5)		17.4 (7.9)
Zinc, mg	11.9 (6.6)	11.1(6.5)	10.4 (5.9)		9.5 (3.6)
Copper, mg	2.5 (1.6)	2.2 (1.5)	2.7 (1.9)		1.8 (1.2)
Manganese, mg	6.5 (3.9)	8.2 (4.8)	12.4 (7.5)		4.9 (2.0)

Abbreviations: FFQ, food frequency questionnaire; 24HRs, 3-day 24-hour dietary recalls; FCT, Food Composition Table;

^a^ FCT1 (WFCT) was calculated for each food group based on the weighted mean of the top 80% of food items by intake amount

^b^ FCT2 (Reference method) was calculated for each food group based on food items provided in previous studies conducted among participants with similar dietary cultures

^c^ FCT3 (‘All food item’ method) was calculated for each food group based on the arithmetic mean of all food items in CFCT

The results of the absolute average intake of nutrients (the WFCT approach) are shown in Table 2. In general, the average daily absolute intakes assessed by FFQ were higher when compared to those assessed by 24HRs, except for total fat. The SCC between the unadjusted nutrients intakes between FFQ and 24HRs ranged from 0.23 for vitamin A to 0.43 for phosphorus. The two energy-adjusted methods attenuated the correlation coefficient for almost all the nutrients except carbohydrates, fiber, and potassium. For example, correlations for iron (unadjusted SCC: 0.29; residual method: 0.16; energy density method: 0.18), zinc (0.40; 0.26; 0.23), and total fat (0.41; 0.26; 0.31) were attenuated after energy-adjustment. The energy-adjusted SCC ranged from 0.16 (iron) to 0.37 (potassium) for the residual method and 0.18 (iron) to 0.38 (carbohydrate) for the energy density method (Table 3).

**Table 3:** Spearman correlation coefficients of daily nutrient intakes between FFQ and 24HRs

Nutrient	FCT1^a^				FCT2^b^				FCT3^c^		
	Unadjusted	Energy-adjusted			Unadjusted	Energy-adjusted			Unadjusted	Energy-adjusted	
		Residual method	Energy Density			Residual method	Energy Density			Residual method	Energy Density
Total energy, kcal	0.38				0.37				0.37		
Protein, g	0.41	0.36	0.35		0.41	0.32	0.31		0.41	0.36	0.35
Total fat, g	0.41	0.26	0.31		0.40	0.27	0.31		0.40	0.23	0.28
Carbohydrate, g	0.29	0.33	0.38		0.29	0.33	0.38		0.27	0.28	0.35
Fiber, g	0.26	0.27	0.30		0.25	0.25	0.28		0.25	0.26	0.27
Vitamin A, µg	0.23	0.19	0.19		0.25	0.21	0.19		0.24	0.17	0.16
Vitamin B1, mg	0.33	0.30	0.30		0.33	0.25	0.25		0.32	0.21	0.21
Vitamin B2, mg	0.41	0.32	0.33		0.42	0.30	0.31		0.41	0.26	0.30
Vitamin C, mg	0.27	0.26	0.27		0.28	0.26	0.26		0.25	0.20	0.20
Calcium, mg	0.31	0.26	0.26		0.30	0.24	0.24		0.33	0.25	0.24
Phosphorus, mg	0.43	0.36	0.33		0.42	0.32	0.30		0.44	0.34	0.32
Potassium, mg	0.34	0.37	0.37		0.34	0.35	0.35		0.32	0.32	0.32
Sodium, mg	0.29	0.25	0.24		0.21	0.17	0.16		0.27	0.23	0.22
Iron, mg	0.29	0.16	0.18		0.29	0.15	0.16		0.26	0.14	0.17
Zinc, mg	0.40	0.26	0.23		0.41	0.25	0.21		0.41	0.24	0.21
Copper, mg	0.29	0.25	0.27		0.29	0.26	0.27		0.28	0.24	0.25
Manganese, mg	0.32	0.26	0.32		0.28	0.16	0.21		0.18	0.09	0.16
Mean	0.33	0.26	0.29		0.33	0.24	0.26		0.32	0.21	0.25

Abbreviations: FFQ, food frequency questionnaire; 24HRs, 3-day 24-hour dietary recalls; FCT, Food Composition Table;

^a^ FCT1 (WFCT) was calculated for each food group based on the weighted mean of the top 80% of food items by intake amount

^b^ FCT2 (Reference method) was calculated for each food group based on food items provided in previous studies conducted among participants with similar dietary cultures

^c^ FCT3 (‘All food item’ method) was calculated for each food group based on the arithmetic mean of all food items in CFCT

## Discussion

We evaluated the performance of a 64-item Chinese FFQ by comparison with 24HRs over three consecutive days among 2325 adults in eastern China and identified the optimal method of developing the FFQ FCT. In general, the average intakes of most food groups and nutrients assessed with FFQ were higher than those assessed using the 24HRs. The correlations were generally attenuated when food and nutrient intakes were adjusted for total energy intake. For food intakes, the average energy-adjusted correlation between FFQ and 24HRs was 0.27, ranging from 0.14 for starch-rich beans to 0.49 for aquatic products. In terms of nutrient assessment, the WFCT for the study FFQ utilizing weights calculated based on the 24HRs data performs the best, with the energy-adjusted SCC ranging from 0.16 for iron to 0.37 for potassium (mean *r* = 0.26).

In a previous meta-analysis of the validity of FFQ among Chinese adults compared with 24HRs, the pooled SCC ranged from 0.28 (edible oil) to 0.62 (milk and dairy products) for food groups, and from 0.34 (manganese) to 0.88 (copper) for nutrients [5]. In particular, a previous study evaluated a Chinese FFQ with 149 food items among 300 Chinese adults aged 25–64 years in Jiangsu Province and Beijing municipality; the crude correlation coefficients between the FFQ and six-repeated 3 consecutive days-24HRs ranged from 0.15 (nuts) to 0.90 (liquor) for foods (mean *r* = 0.55) and from 0.22 (vitamin C) to 0.84 (polyunsaturated fatty acid) for nutrients (mean *r* = 0.56) [19]. In another two validation studies executed in the Shanghai Men’s Health Study (SMHS) and Shanghai Women’s Health Study (SWHS), the investigators evaluated an 81-food-item FFQ with multiple 24HRs in Shanghai residents aged 40–70 years [20, 21]. The crude correlations of food groups for the SWHS and the SMHS ranged from 0.38 (all vegetables) to 0.65 (rice) (mean *r* = 0.49), and from 0.35 (poultry) to 0.72 (fruits) (mean *r* = 0.50), respectively. Correlations of nutrients ranged from 0.41 (carotene) to 0.66 (carbohydrates) (mean *r* = 0.50) for the SWHS, and from 0.33 (retinol) to 0.64 (carbohydrates) (mean *r* = 0.47) for the SMHS [20, 21]. In the current study, we evaluated the 64-food-item FFQ with one 3-day 24HRs, the weaker correlations were found for the food groups of starch-rich beans, potatoes, nuts and for the nutrients of vitamin A and vitamin C. The stronger correlations were found for the food groups of fruits, red meat, poultry, dairy and its products and for the nutrients of total fat, protein, phosphorus, and vitamin B2. In general, although the average correlations in our study were lower compared to previous studies, the overall correlations trends were consistent with previous studies. Of note, the discrepancy between previous studies and our findings suggests that the 64-item FFQ in the current study might benefit by adding regional-specific foods and covering more food items to provide more accurate dietary information.

In the Shanghai Diet and Health Study, investigators reported a method of nutrients calculation depending on the data of 24HRs and weighted by the constituent ratio of the amount of the top 10 food items in each food group (‘top 10’ method). The SCC for the nutrient intakes ranged from 0.33 (zinc) to 0.77 (carbohydrates), indicating a relatively good validity [6]. In our study, we evaluated eight converted FCTs including the ‘top 10’ method, and the results for each FCT were relatively similar. The nutrient validity correlation coefficient ranged from 0.23 (vitamin A) to 0.43 (phosphorus) for the ‘top 10’ methods, but the performance was slightly lower for the nutrients of sodium and manganese compared to the WFCT method in our study. Overall, despite heterogeneity exist across the current study and previous studies, which might due to the inconsistencies in the number of FFQ food items, reference methods, and study period, our FFQ could provide valuable nutritional information with the WFCT. The current study was a large-scale study to evaluate the validity of FFQ with varying converted FCT established based on professional knowledge and representative 24HRs data among the eastern China population. However, it had several limitations. First, the 3-day 24HRs was assessed only once in our study, thus we couldn’t correct for its random error and underestimated the long-term validity of the FFQ. Second, there were not nutritional biomarkers in this study, thus we could not evaluate the FFQ validity with biomarkers as the reference method. Third, 24HRs would have memory bias, however, many previous studies used this method as a reference method to evaluate the validity of FFQ. Fourth, our validity results might not be generalizable to populations in other regions with different dietary cultures [22–24].

In conclusion, the 64-item Chinese FFQ and the WFCT were reasonably valid to assess the dietary intake of foods and nutrients among adults in eastern China. As a structured instrument to assess long-term habitual diet, the FFQ has the potential to make contributions to the population-based research of nutrition and health in China.

### Electronic supplementary material

Below is the link to the electronic supplementary material.

**Supplementary Material 1:** Supplementary material

## Data Availability

Data described in the manuscript, code book, and analytic code will not be made publicly available due to privacy and ethical restrictions.

## Abbreviations

BMI: body mass index
CACDNS: China Adults Chronic Disease and Nutrition Surveillance
CFCT: Chinese FCT
FCT: food composition table
FFQ: food frequency questionnaire
SCC: Spearman correlation coefficients
SD: standard deviation
SMHS: Shanghai Men’s Health Study
SWHS: Shanghai Women’s Health Study
WFCT: weighted FCT
24HRs: 3-day 24-hour recalls
