# Supplementary material for: Validity of food and nutrient intakes assessed by a food frequency questionnaire among Chinese adults
Source: Nutr J. 2024 Feb 27;23:23. doi: 10.1186/s12937-024-00921-9 (PMC10898109; doi:10.1186/s12937-024-00921-9)
Supplement: Supplementary file 1 — Supplementary Material 1: Supplementary material [file 12937_2024_921_MOESM1_ESM.docx]

**Validity of food and nutrient intakes assessed by a Food Frequency Questionnaire among Chinese adults**

Dong Zhao et al

**Online Supplementary Material**

**Tables**

**Supplementary Table 1** The generation methods of eight food composition tables (FCTs)

**Supplementary Table 2** Absolute daily foods consumption estimated by FFQ and 24HRs

**Supplementary Table 3** Spearman correlations coefficients of daily foods consumption between FFQ and 24HRs

**Supplementary Table 4** Mean values and standard deviations for absolute daily nutrient intakes estimated by FFQ generated by different FCTs

**Supplementary Table 5** Spearman correlation coefficients of daily nutrient intakes between FFQ generated by different FCT and 24HRs

**Supplementary Table1:** The generation methods of eight food composition tables (FCTs)

| **FCT name** | **Generation methods** |
| --- | --- |
| FCT1: the WFCT | 1. Rank the food items of each food group in accordance with their intake amount from high to low based on 3 day 24 hour recalls (24HR) data; 2. Select the top 80% food items of each food group; 3. Generate the FCT1(the WFCT) by calculating the weighted mean of the selected food items and weighted by their constituent ratios of total amount in each food group. |
| FCT2: the reference method | 1. Select the food items of each food group with dietary cultures similar to Zhejiang residents in previous studies; 2. Generate the FCT2 (the reference method) by calculating the arithmetic mean of the selected food items in each food group. |
| FCT3: ‘all food items’ method | 1. Select all the food items in the 2002 Chinese FCT of each food group; 2. Generate the FCT3 (‘all food items’ method) by calculating the arithmetic mean of the selected food items in each food group. |
| FCT4 | 1. Rank the food items of each food group in accordance with their intake amount from high to low based on 3 day 24 hour recalls (24HR) data; 2. Select the top 80% food items of each food group; 3. Generate the FCT4 by calculating the arithmetic mean of the selected food items in each food group. |
| FCT5 | 1. Rank the food items of each food group in accordance with their intake frequency from high to low based on 3 day 24 hour recalls (24HR) data; 2. Select the top 80% food items of each food group; 3. Generate the FCT5 by calculating the arithmetic mean of the selected food items in each food group. |
| FCT6 | 1. Rank the food items of each food group in accordance with their intake frequency from high to low based on 3 day 24 hour recalls (24HR) data; 2. Select the top 80% food items of each food group; 3. Generate the FCT6 by calculating the weighted mean of the selected food items and weighted by their constituent ratios of total amount in each food group. |
| FCT7 | 1. Rank the food items of each food group in accordance with their intake amount from high to low based on 3 day 24 hour recalls (24HR) data; 2. Select the top 10 food items of each food group; 3. Generate the FCT7 by calculating the arithmetic mean of the selected food items in each food group. |
| FCT8 | 1. Rank the food items of each food group in accordance with their intake amount from high to low based on 3 day 24 hour recalls (24HR) data; 2. Select the top 10 food items of each food group; 3. Generate the FCT8 by calculating the weighted mean of the selected food items and weighted by their constituent ratios of total amount in each food group. |

| **Supplementary Table 2.** Absolute daily foods consumption estimated by FFQ and 24HRs | | | | | | | | | | | |
| --- | --- | --- | --- | --- | --- | --- | --- | --- | --- | --- | --- |
| **Food Groups** | **24HRs** | | | | |  | **FFQ** | | | | |
|  | **Mean** | **SD** | **Median** | **P25** | **P75** |  | **Mean** | **SD** | **Median** | **P25** | **P75** |
| Cereals(g) | 217.3 | 97.7 | 200.0 | 145.5 | 275.0 |  | 352.4 | 231.3 | 303.6 | 210.0 | 442.9 |
| Potatoes(g) | 20.3 | 38.4 | 0.0 | 0.0 | 33.3 |  | 16.4 | 31.7 | 6.7 | 1.7 | 17.3 |
| Starch-rich Beans(g) | 4.1 | 15.7 | 0.0 | 0.0 | 0.0 |  | 2.3 | 7.8 | 0.0 | 0.0 | 1.7 |
| Soybean(g)&its products(g) | 24.6 | 35.6 | 11.6 | 0.0 | 33.3 |  | 18.1 | 31.9 | 10.8 | 4.3 | 22.2 |
| Vegetables(g) | 261.8 | 157.1 | 233.3 | 150.0 | 336.5 |  | 333.5 | 266.7 | 280.0 | 158.1 | 425.6 |
| Fruits(g) | 42.1 | 69.6 | 0.0 | 0.0 | 66.7 |  | 90.3 | 137.6 | 45.7 | 14.3 | 107.1 |
| Nuts(g) | 4.6 | 14.2 | 0.0 | 0.0 | 0.0 |  | 5.4 | 15.6 | 0.7 | 0.0 | 5.0 |
| Red meat & Poultry(g) | 80.9 | 71.4 | 66.7 | 33.3 | 113.3 |  | 62.0 | 75.7 | 42.9 | 20.0 | 82.1 |
| Dairy & its products(g) | 14.5 | 48.4 | 0.0 | 0.0 | 0.0 |  | 45.5 | 151.3 | 0.0 | 0.0 | 28.6 |
| Eggs(g) | 17.8 | 30.0 | 8.8 | 0.0 | 26.7 |  | 27.7 | 33.2 | 20.1 | 8.6 | 37.4 |
| Aquatic products(g) | 56.5 | 76.6 | 33.3 | 0.0 | 81.0 |  | 53.9 | 93.9 | 24.5 | 7.2 | 62.2 |
| Beverages(g) | 6.7 | 42.7 | 0.0 | 0.0 | 0.0 |  | 21.3 | 62.2 | 0.0 | 0.0 | 13.3 |
| Liquors(g) | 4.3 | 12.4 | 0.5 | 0.0 | 2.2 |  | 11.1 | 36.7 | 0.0 | 0.0 | 2.0 |
| Desserts(g) | 7.2 | 20.3 | 0.0 | 0.0 | 0.0 |  | 12.8 | 29.3 | 2.2 | 0.0 | 14.3 |

Abbreviations: FFQ, food frequency questionnaire; 24HRs, 3-day 24-hour dietary recalls; SD, standard deviation; P,percentage

| **Supplementary Table 3.** Spearman correlations coefficients of daily foods consumption between FFQ and 24HRs | | | |
| --- | --- | --- | --- |
| **Food Groups** | **Unadjusted** | **Energy-adjusted** | |
|  |  | **Residual method** | **Energy Density** |
| Cereals(g) | 0.33 | 0.36 | 0.39 |
| Potatoes(g) | 0.15 | 0.16 | 0.17 |
| Starch-rich Beans(g) | 0.11 | 0.14 | 0.11 |
| Soybean(g)&its products(g) | 0.23 | 0.17 | 0.22 |
| Vegetables(g) | 0.29 | 0.27 | 0.29 |
| Fruits(g) | 0.45 | 0.43 | 0.44 |
| Nuts(g) | 0.16 | 0.16 | 0.15 |
| Red meat & Poultry(g) | 0.44 | 0.26 | 0.35 |
| Dairy & its products(g) | 0.42 | 0.35 | 0.42 |
| Eggs(g) | 0.28 | 0.23 | 0.23 |
| Aquatic products(g) | 0.56 | 0.49 | 0.54 |
| Beverages(g) | 0.24 | 0.16 | 0.23 |
| Liquors(g) | 0.36 | 0.38 | 0.34 |
| Desserts(g) | 0.23 | 0.19 | 0.22 |
| Mean | 0.30 | 0.27 | 0.29 |

Abbreviations: FFQ, food frequency questionnaire; 24HRs, 3-day 24-hour dietary recalls.

**Supplementary Table 4.** Mean values and standard deviations for absolute daily nutrient intakes estimated by FFQ generated by different FCT

| **Nutrient** | **FFQ** | | | | |
| --- | --- | --- | --- | --- | --- |
|  | **FCT4 ^a^** | **FCT5 ^b^** | **FCT6 ^c^** | **FCT7 ^d^** | **FCT8 ^e^** |
| Total energy, kcal | 1,921.4 (1,084.6) | 1,957.1 (1,098.8) | 1,948.4 (1,100.9) | 1,744.0 (998.1) | 1,918.1 (1,088.4) |
| Protein, g | 71.8 (44.9) | 71.3 (44.6) | 70.2 (44.0) | 71.0 (45.1) | 69.8 (43.9) |
| Total fat, g | 29.6 (24.4) | 33.3 (28.3) | 34.0 (29.8) | 30.9 (25.4) | 33.9 (29.8) |
| Carbohydrate, g | 327.7 (191.4) | 329.6 (191.5) | 326.1 (190.1) | 281.6 (161.0) | 319.3 (186.2) |
| Fiber, g | 12.8 (13.3) | 12.0 (11.5) | 12.0 (11.6) | 12.3 (11.4) | 12.2 (12.8) |
| Vitamin A, µg | 392.6 (268.0) | 473.5 (325.8) | 464.3 (322.7) | 374.7 (254.4) | 550.4 (378.8) |
| Vitamin B1, mg | 1.2 (1.9) | 1.2 (1.9) | 1.2 (1.9) | 1.1 (1.9) | 1.2 (1.9) |
| Vitamin B2, mg | 2.0 (8.9) | 2.0 (8.9) | 2.0 (8.9) | 1.9 (8.9) | 2.0 (8.9) |
| Vitamin C, mg | 91.8 (80.5) | 118.5 (106.6) | 102.9 (89.0) | 100.1 (85.9) | 100.9 (86.3) |
| Calcium, mg | 497.5 (398.6) | 473.3 (351.4) | 495.4 (362.9) | 480.9 (376.2) | 522.9 (397.3) |
| Phosphorus, mg | 1,042.5 (615.3) | 1,017.4 (611.7) | 1,027.6 (619.6) | 921.9 (575.0) | 1,012.6 (608.0) |
| Potassium, mg | 1,850.4 (1,197.1) | 1,878.7 (1,282.1) | 1,866.2 (1,286.1) | 1,768.0 (1,212.3) | 1,800.0 (1,209.8) |
| Sodium, mg | 1,033.0 (1,214.6) | 1,256.7 (1,301.5) | 1,101.8 (1,278.3) | 1,311.9 (1,499.8) | 1,047.2 (1,203.3) |
| Iron, mg | 22.5 (23.8) | 21.4 (19.6) | 21.6 (19.9) | 22.2 (36.1) | 21.9 (22.3) |
| Zinc, mg | 12.2 (6.7) | 12.0 (6.7) | 11.9 (6.7) | 10.1 (5.8) | 11.7 (6.5) |
| Copper, mg | 3.0 (2.8) | 2.7 (2.3) | 2.5 (1.7) | 2.6 (1.9) | 2.4 (1.6) |
| Manganese, mg | 6.6 (3.9) | 7.4 (4.2) | 6.9 (4.1) | 5.5 (3.2) | 6.4 (3.9) |

Abbreviations: FFQ, food frequency questionnaire; FCT, Food Composition Table; CFCT, Chinese Food Composition Table.

^a^ FCT4 was calculated for each food group based on the arithmetic mean of the top 80% of food items by intake amount.

^b^ FCT5 was calculated for each food group based on the arithmetic mean of the top 80% of food items by intake frequency.

^c^ FCT6 was calculated for each food group based on the weighted mean of the top 80% of food items by intake frequency.

^d^ FCT7 was calculated for each food group based on the arithmetic mean of the top 10 food items by intake amount.

^e^ FCT8 was calculated for each food group based on the weighted mean of the top 10 food items by intake amount.

**Supplementary Table 5.** Spearman correlation coefficients of daily nutrient intakes between FFQ generated by different FCT and 24HRs

| **Nutrient** | **FCT4 ^a^** | | |  | **FCT5 ^b^** | | |  | **FCT6 ^c^** | | | |
| --- | --- | --- | --- | --- | --- | --- | --- | --- | --- | --- | --- | --- |
|  | **Unadjusted** | **Energy-adjusted** | |  | **Unadjusted** | **Energy-adjusted** | |  | **Unadjusted** | **Energy-adjusted** | | |
|  |  | **Residual method** | **Energy Density** |  |  | **Residual method** | **Energy Density** |  |  | **Residual method** | | **Energy Density** |
| Total energy, kcal | 0.37 |  |  |  | 0.37 |  |  |  | 0.37 |  |  | |
| Protein, g | 0.41 | 0.35 | 0.34 |  | 0.41 | 0.35 | 0.34 |  | 0.41 | 0.36 | 0.34 | |
| Total fat, g | 0.41 | 0.27 | 0.31 |  | 0.41 | 0.27 | 0.31 |  | 0.41 | 0.26 | 0.31 | |
| Carbohydrate, g | 0.29 | 0.33 | 0.38 |  | 0.29 | 0.32 | 0.38 |  | 0.29 | 0.32 | 0.38 | |
| Fiber, g | 0.26 | 0.28 | 0.29 |  | 0.26 | 0.28 | 0.30 |  | 0.26 | 0.27 | 0.29 | |
| Vitamin A, µg | 0.24 | 0.22 | 0.20 |  | 0.23 | 0.20 | 0.19 |  | 0.24 | 0.20 | 0.19 | |
| Vitamin B1, mg | 0.33 | 0.26 | 0.26 |  | 0.33 | 0.28 | 0.28 |  | 0.33 | 0.29 | 0.29 | |
| Vitamin B2, mg | 0.41 | 0.30 | 0.31 |  | 0.41 | 0.30 | 0.31 |  | 0.41 | 0.30 | 0.31 | |
| Vitamin C, mg | 0.28 | 0.26 | 0.27 |  | 0.28 | 0.26 | 0.26 |  | 0.28 | 0.26 | 0.27 | |
| Calcium, mg | 0.31 | 0.25 | 0.25 |  | 0.32 | 0.26 | 0.26 |  | 0.31 | 0.26 | 0.26 | |
| Phosphorus, mg | 0.43 | 0.35 | 0.32 |  | 0.43 | 0.35 | 0.33 |  | 0.43 | 0.35 | 0.33 | |
| Potassium, mg | 0.34 | 0.36 | 0.36 |  | 0.34 | 0.36 | 0.36 |  | 0.34 | 0.36 | 0.36 | |
| Sodium, mg | 0.29 | 0.25 | 0.24 |  | 0.26 | 0.22 | 0.22 |  | 0.28 | 0.24 | 0.23 | |
| Iron, mg | 0.29 | 0.16 | 0.17 |  | 0.29 | 0.16 | 0.18 |  | 0.29 | 0.16 | 0.18 | |
| Zinc, mg | 0.41 | 0.24 | 0.21 |  | 0.41 | 0.25 | 0.23 |  | 0.40 | 0.25 | 0.23 | |
| Copper, mg | 0.27 | 0.22 | 0.23 |  | 0.28 | 0.24 | 0.25 |  | 0.28 | 0.25 | 0.26 | |
| Manganese, mg | 0.31 | 0.26 | 0.32 |  | 0.30 | 0.22 | 0.28 |  | 0.31 | 0.24 | 0.30 | |
| Mean | 0.33 | 0.26 | 0.28 |  | 0.33 | 0.25 | 0.28 |  | 0.33 | 0.26 | 0.28 | |

Abbreviations: FFQ, food frequency questionnaire; 24HRs, 3-day 24-hour dietary recalls; FCT, Food Composition Table; CFCT, Chinese Food Composition Table.

^a^ FCT4 was calculated for each food group based on the arithmetic mean of the top 80% of food items by intake amount.

^b^ FCT5 was calculated for each food group based on the arithmetic mean of the top 80% of food items by intake frequency.

^c^ FCT6 was calculated for each food group based on the weighted mean of the top 80% of food items by intake frequency.

**(Continued) Supplementary Table 5** Spearman correlation coefficients of daily nutrient intakes between FFQ generated by different FCT and 24HRs

| **Nutrient** | **FCT7^d^** | | |  | **FCT8^e^** | | |
| --- | --- | --- | --- | --- | --- | --- | --- |
|  | **Unadjusted** | **Energy-adjusted** | |  | **Unadjusted** | **Energy-adjusted** | |
|  |  | **Residual method** | **Energy Density** |  |  | **Residual method** | **Energy Density** |
| Total energy, kcal | 0.37 |  |  |  | 0.38 |  |  |
| Protein, g | 0.41 | 0.33 | 0.32 |  | 0.41 | 0.36 | 0.34 |
| Total fat, g | 0.41 | 0.26 | 0.30 |  | 0.41 | 0.26 | 0.31 |
| Carbohydrate, g | 0.28 | 0.31 | 0.38 |  | 0.29 | 0.32 | 0.38 |
| Fiber, g | 0.26 | 0.27 | 0.29 |  | 0.26 | 0.27 | 0.29 |
| Vitamin A, µg | 0.24 | 0.19 | 0.18 |  | 0.23 | 0.19 | 0.18 |
| Vitamin B1, mg | 0.33 | 0.24 | 0.24 |  | 0.33 | 0.29 | 0.29 |
| Vitamin B2, mg | 0.41 | 0.26 | 0.30 |  | 0.41 | 0.30 | 0.31 |
| Vitamin C, mg | 0.27 | 0.25 | 0.27 |  | 0.27 | 0.26 | 0.27 |
| Calcium, mg | 0.32 | 0.25 | 0.26 |  | 0.31 | 0.26 | 0.26 |
| Phosphorus, mg | 0.42 | 0.34 | 0.33 |  | 0.43 | 0.35 | 0.33 |
| Potassium, mg | 0.34 | 0.35 | 0.35 |  | 0.34 | 0.37 | 0.37 |
| Sodium, mg | 0.27 | 0.22 | 0.22 |  | 0.28 | 0.25 | 0.24 |
| Iron, mg | 0.30 | 0.16 | 0.18 |  | 0.29 | 0.16 | 0.18 |
| Zinc, mg | 0.41 | 0.25 | 0.23 |  | 0.40 | 0.22 | 0.23 |
| Copper, mg | 0.28 | 0.24 | 0.25 |  | 0.29 | 0.25 | 0.27 |
| Manganese, mg | 0.29 | 0.24 | 0.30 |  | 0.31 | 0.26 | 0.32 |
| Mean | 0.33 | 0.23 | 0.27 |  | 0.33 | 0.26 | 0.29 |

Abbreviations: FFQ, food frequency questionnaire; 24HRs, 3-day 24-hour dietary recalls; FCT, Food Composition Table; CFCT, Chinese Food Composition Table.

^d^ FCT7 was calculated for each food group based on the arithmetic mean of the top 10 food items by intake amount.

^e^ FCT8 was calculated for each food group based on the weighted mean of the top 10 food items by intake amount.
